# Supplementary figures and images for: GeneTEFlow: A Nextflow-based pipeline for analysing gene and transposable elements expression from RNA-Seq data
Source: PLoS One. 2020 Aug 31;15(8):e0232994. doi: 10.1371/journal.pone.0232994 (PMC7458328; doi:10.1371/journal.pone.0232994)

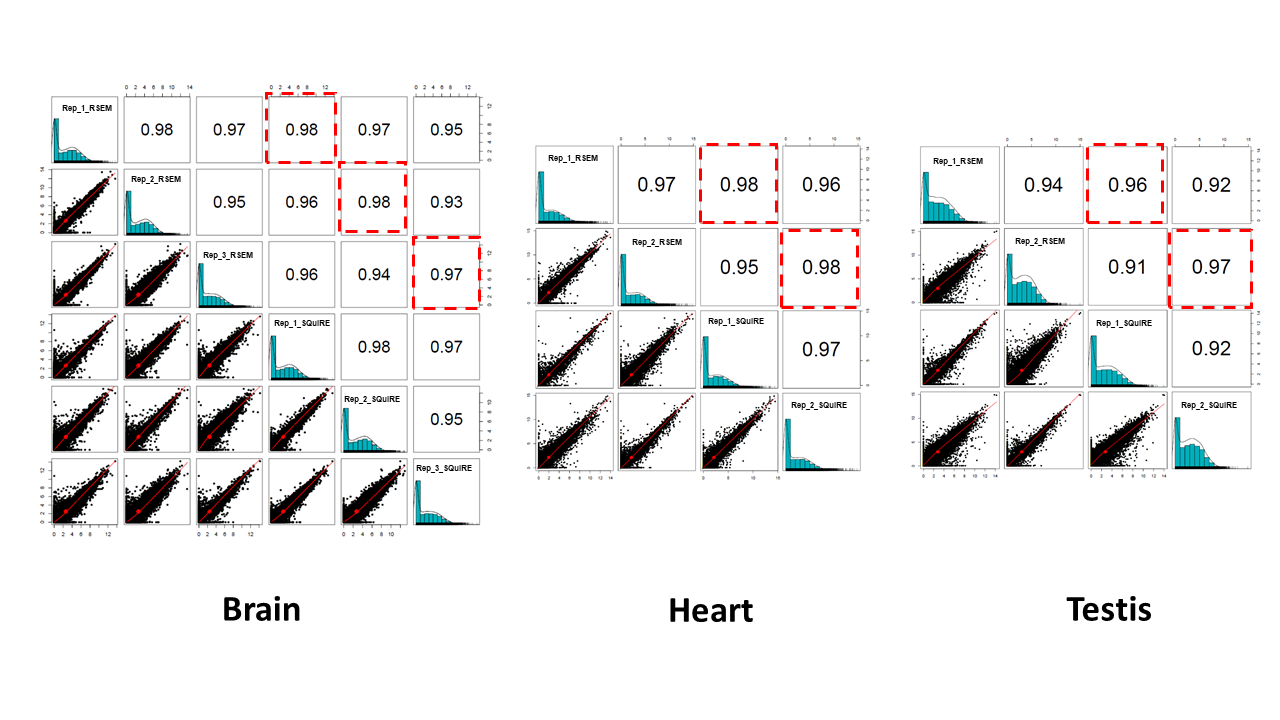

Supplement: S1 Fig — Gene expression (total 22,955 genes) of samples from brain tissues (left), heart tissues (middle), and testis tissues (right) was calculated by both RSEM and SQuIRE. Lower diagonal panels: pairwise comparisons using log2(TPM + 1) of 22,955 genes. Upper diagonal panels: Pearson correlation coefficient of each comparison. Panels highlighted in red: Pearson correlation coefficient of comparisons between RSEM and SQuIRE gene expression quantification of the same sample. Rep_: replicate, _RSEM: quantification performed by RSEM, _SQuIRE: quantification performed by SQuIRE. (TIF) [file pone.0232994.s001.tif]
