# Supplementary material for: GeneTEFlow: A Nextflow-based pipeline for analysing gene and transposable elements expression from RNA-Seq data
Source: PLoS One. 2020 Aug 31;15(8):e0232994. doi: 10.1371/journal.pone.0232994 (PMC7458328; doi:10.1371/journal.pone.0232994)
Supplement: S1 Table — (DOCX) [file pone.0232994.s002.docx]

**S1 Table.** Major bioinformatics tools installed in GeneTEFlow

| **Tools or packages** | **Version** |
| --- | --- |
| 1. **Raw reads pre-processing and quality control** |  |
| Trimmomatic | v0.36 |
| FastQC | v0.11.7 |
| 1. **Reads alignment** |  |
| STAR | v2.6.0c |
| HISAT2 | v2.1.0 |
| 1. **Gene quantification** |  |
| RSEM | v1.3.0 |
| StringTie | v1.3.4 |
| 1. **Differential expression analysis** |  |
| DESeq2 | v1.18.1 |
| **RNASeq analysis for transposable elements** |  |
| 1. SQuIRE | v1.0 |
| **Gene set enrichment analysis** |  |
| 1. GSEA | v3.0 |
| 1. **Other tools and in-house analysis scripts** |  |
| samtools | v1.3.1 |
| bedtools | v2.25.0 |
| 1. R | v3.4.1 |
| 1. ggplot2 | v3.0.0 |
| 1. Venndiagram | v1.6.20 |
| 1. Complexheatmap | v1.18.1 |
| Tximport | v1.8.0 |
| In-house Rscripts | v1.0 |
