# Supplementary material for: GeneTEFlow: A Nextflow-based pipeline for analysing gene and transposable elements expression from RNA-Seq data
Source: PLoS One. 2020 Aug 31;15(8):e0232994. doi: 10.1371/journal.pone.0232994 (PMC7458328; doi:10.1371/journal.pone.0232994)
Supplement: S2 Table — (DOCX) [file pone.0232994.s003.docx]

**S2 Table.** Major outputs from GeneTEFlow

| **Output files** | **Format** |
| --- | --- |
| **Raw reads quality control** |  |
| FastQC output | HTML |
| **Quality control after reads alignment** |  |
| SampleQC | PDF |
| **Quantification** |  |
| all.sample.Counts.genes.results | TXT |
| all.sample.TPM.genes.results | TXT |
| all.sample.Counts.TE.results | TXT |
| all.sample.TPM.TE.results | TXT |
| **Differential expression analysis** |  |
| all.deseq2.genes.out.txt | TXT |
| all.deseq2.TE.out.txt | TXT |
| **Summary report** |  |
| all.sample.siggene.sum.txt | TXT |
| all.sig.gene.subset.*sum.pdf | PDF |
| all.sample.sigTE.sum.txt | TXT |
| all.sig.TE.subset.*sum.pdf | PDF |
| **Gene set enrichment analysis** |  |
| GSEA output | ZIP file |
| **Pipeline logs** |  |
| nf.report.html | HTML |
| nf.timeline.html | HTML |
