# Supplementary material for: GeneTEFlow: A Nextflow-based pipeline for analysing gene and transposable elements expression from RNA-Seq data
Source: PLoS One. 2020 Aug 31;15(8):e0232994. doi: 10.1371/journal.pone.0232994 (PMC7458328; doi:10.1371/journal.pone.0232994)
Supplement: S3 Table — (DOCX) [file pone.0232994.s004.docx]

**S3 Table.** Human RNA-Seq data used in the example application of GeneTEFlow

| **Samples** | **GEO number** | **SRR number** |
| --- | --- | --- |
| Brain replicate 1 | GSM752691 | SRR306838 |
| Brain replicate 2 | GSM752694 | SRR306841 |
| Brain replicate 3 | GSM752692 | SRR306839 |
| Heart replicate 1 | GSM752699 | SRR306847 |
| Heart replicate 2 | GSM752701 | SRR306850 |
| Testis replicate 1 | GSM752707 | SRR306857 |
| Testis replicate 2 | GSM752708 | SRR306858 |
